# Unveiling heterogeneity and prognostic markers in ductal breast cancer through single-cell RNA-seq

**DOI:** 10.1186/s12935-024-03325-1

**Published:** 2024-07-27

**Authors:** Jianxun Hou, Wei Liu, Meihong Yan, Yanlv Ren, Cheng Qian, Yingqiang Fu, Hongbin Wang, Zhigao Li

**Affiliations:** 1 The Second Department of Breast Surgery, Harbin Medical University Cancer Hospital, No. 150, Haping Road, Nangang District, Harbin, Heilongjiang Province 150081 P. R. China; 2 The Fourth Department of Medical Oncology, Harbin Medical University Cancer Hospital, Harbin, 150081 P. R. China

**Keywords:** Ductal breast cancer, Cellular heterogeneity, Single-cell RNA-seq, Lasso regression, CYP24A1, TFPI2

## Abstract

**Background:**

Breast cancer (BC) is a heterogeneous disease, with the ductal subtype exhibiting significant cellular diversity that influences prognosis and response to treatment. Single-cell RNA sequencing data from the GEO database were utilized in this study to investigate the underlying mechanisms of cellular heterogeneity and to identify potential prognostic markers and therapeutic targets.

**Methods:**

Bioinformatics analysis was conducted using R packages to analyze the single-cell sequencing data. The presence of highly variable genes and differences in malignant potency within the same BC samples were examined. Differential gene expression and biological function between Type 1 and Type 2 ductal epithelial cells were identified. Lasso regression and Cox proportional hazards regression analyses were employed to identify genes associated with patient prognosis. Experimental validation was performed in vitro and in vivo to confirm the functional relevance of the identified genes.

**Results:**

The analysis revealed notable heterogeneity among BC cells, with the presence of highly variable genes and differences in malignant behavior within the same samples. Significant disparities in gene expression and biological function were identified between Type 1 and Type 2 ductal epithelial cells. Through regression analyses, CYP24A1 and TFPI2 were identified as pivotal genes associated with patient prognosis. Kaplan-Meier curves demonstrated their prognostic significance, and experimental validation confirmed their inhibitory effects on malignant behaviors of ductal BC cells.

**Conclusion:**

This study highlights the cellular heterogeneity in ductal subtype breast cancer and delineates the differential gene expressions and biological functions between Type 1 and Type 2 ductal epithelial cells. The genes CYP24A1 and TFPI2 emerged as promising prognostic markers and therapeutic targets, exhibiting inhibitory effects on BC cell malignancy in vitro and in vivo. These findings offer the potential for improved BC management and the development of targeted treatment strategies.

**Supplementary Information:**

The online version contains supplementary material available at 10.1186/s12935-024-03325-1.

## Background

Breast cancer persistently poses an extensive health dilemma globally, notably afflicting women, especially within developed countries [1]. The advancement in detecting and managing breast cancer has been noteworthy; however, patient survival remains jeopardized due to persistent challenges related to recurrence and metastatic events [2]. The necessity to delve into breast cancer’s molecular and biological complexity is paramount, aiming to decode its multifaceted nature and subsequently ushering in specialized therapeutic strategies [1]. Tumor cell heterogeneity has emerged as a focal point in recent research due to its crucial role in influencing tumor growth, metastasis, and therapeutic response.

The heterogeneity among tumor cells signifies the existence of distinct molecular and biological characteristics within the same tumor, emanating from various factors such as genetic mutations, epigenetic modifications, and interactions with the tumor microenvironment [3, 4]. Ductal breast cancer, a prevalent subtype, has become a notable entity for exploring cellular heterogeneity and its implications for disease progression and management [5]. Traditional Bulk RNA-seq has been instrumental in mapping the genetic landscape of tumors, yet its capacity to delineate individual cellular variations is limited [6, 7]. Conversely, single-cell RNA-seq, a burgeoning technique, enables meticulous gene expression profiling at the single-cell level, thereby providing a robust platform to navigate through the complexity of tumor cell heterogeneity [8].

The application of single-cell RNA-seq enables a granular exploration of gene expression within diverse cellular populations within breast cancer tissue, allowing for the illumination of pivotal genes that may modulate tumor behavior and patient prognosis [9]. The genes thus identified could serve as potential biomarkers for patient management and further research into prospective therapeutic interventions, propelling the development of more nuanced and individualized treatment plans for those affected by breast cancer [10].

This research venture, set within the aforementioned context, aspires to explore the molecular distinctions and heterogeneity within ductal breast cancer cells in a nuanced manner. Utilizing single-cell RNA-seq as a sophisticated screening modality, it aims to identify and validate prognostic molecular markers. The broader objective encompasses not merely enhancing our understanding of the mechanistic underpinnings of breast cancer but also translating such insights into clinically relevant interventions, aiming to uplift survival outcomes and enrich the quality of life for those navigating through the complexities of this disease.

## Materials and methods

### Data retrieval

Bulk RNA-seq data and clinical information for the BC (TCGA-BC) cohort were downloaded from the UCSC Xena browser (https://xenabrowser.net/). Single-cell RNA-seq data for BC (GSE118389) were downloaded from the Gene Expression Omnibus (GEO) database (http://www.ncbi.nlm.nih.gov/geo/); chip data samples for GSE118389 were obtained from tumor tissues of 6 BC patients, and the sequencing libraries were constructed using an optimized Smart-seq2 method, followed by sequencing on Illumina NextSeq 500. The raw single-cell expression matrix for 1534 cells provided by the GEO database was downloaded. All analyses in this study were conducted using R software, version v3.6.1.

### Single-cell RNA-seq analysis

An integration of single-cell transcriptome Figure spectra for downstream analysis was conducted. The R package Seurat (https://satijalab.org/seurat, version 2.2) [11] was employed for the analysis of single-cell RNA-seq and the mitigation of batch effects within the data. The t-SNE algorithm was utilized for non-linear dimensionality reduction of the single-cell sequencing data. Clustering of individual cells, identification of marker genes for each cluster, and exportation of matrices with unique molecular identifier (UMI) values for each gene in individual cells were accomplished using Cell Ranger. Marker genes for various cell clusters were identified through the Seurat package. The majority of the Seurat analyses were conducted using default parameters. For the FeaturePlot function, a max cutoff of 0.5 was employed. Annotation of cell clusters was achieved using the SingleR package [12]. The R package “inferCNV” (https://github.com/broadinstitute/inferCNV) was utilized for the analysis of single-cell copy number variations).

### GO enrichment analysis

The ClusterProfiler R package [13] was employed to conduct GO (Gene Ontology) enrichment analysis on differentially expressed genes, with an adjustment made for gene length bias. GO terms were deemed significantly enriched by the differentially expressed genes if they exhibited an adjusted p-value below 0.05.

### Survival analysis

The “Survival” R package (https://CRAN.R-project.org/package=Survival) was utilized for survival analysis of risk scores. Wald statistics, generated through univariate Cox proportional hazards regression, could be allocated to each gene as weights. Risk scores for each patient were computed using a linear combination of weighted gene expression (Hazard Ratio, HR) [14]. Overall survival was analyzed through the Kaplan-Meier method. Diagnostic ROC based on the breast cancer dataset from the TCGA database was performed using the “pROC” R package (https://cran.r-project.org/web/packages/pROC/index.html), and visualization of figures was conducted using the “ggplot2” package. The statistical significance of survival differences was tested through the log-rank test. A P-value less than 0.05 was considered to indicate statistical significance.

### Clinical sample collection

Biopsy specimens from 35 breast cancer (BC) patients were collected from our institution, with adjacent non-cancerous tissue serving as the normal control. No patients had undergone radiation or chemotherapy prior to surgery. All samples were classified and graded according to the WHO histological classification of breast cancer and confirmed by pathology. The study was approved by our institution’s ethical review committee. Informed consent was obtained from all patients, adhering to the Declaration of Helsinki.

### Cell culture and transfection

Normal human mammary epithelial cells MCF-10 A and breast cancer (BC) cell lines T47D and MCF-7 were purchased from the ATCC collection. All the aforementioned cells were cultured in DMEM (10,569,044, Gibco, USA) supplemented with 10% FBS (100,099,141, Gibco, USA) and 1% penicillin-streptomycin (15,070,063, Gibco, USA) and were maintained in an incubator at 37 °C with 5% CO2.

MCF-7 cells, when in the logarithmic phase, were digested with trypsin and then seeded in a 6-well plate at a density of 1 × 105 cells per well. Conventional cultivation was continued for 24 h, and once the cell confluence reached approximately 40%, the cells underwent lentivirus infection according to the lentivirus infection instructions. The cells were divided into three groups: oe-NC group (infected with empty vector control lentivirus, oe-NC), oe-CYP24A1 group (infected with lentivirus oe-CYP24A1), and oe-TFPI2 group (infected with lentivirus oe-TFPI2). Cells were harvested for subsequent experiments 72 h post-transfection.

### RT-qPCR

Total RNA from tissues or cells was isolated utilizing Trizol (16,096,020, Thermo Fisher Scientific, New York, USA). For mRNA detection, reverse transcription was executed with a reverse transcription kit (RR047A, Takara, Japan) to produce cDNA. Utilizing the SYBR Premix Ex TaqTM II kit (DRR081, Takara, Japan), sample addition was performed, and qRT-PCR reactions were conducted using a real-time fluorescence quantitative PCR instrument (ABI 7500, ABI, Foster City, CA, USA). GAPDH served as the internal reference gene for coding genes. The PCR program was established as follows: 95 °C for 10 min, followed by 35 cycles of 95 °C for 15 s, 60 °C for 30 s, and 72 °C for 45 s. All qRT-PCR setups were conducted in triplicate. The 2-ΔΔCt method was employed to illustrate the fold-change relationship between the expression of the target gene in the experimental and control groups, using the formula: ΔΔCT = ΔCt experimental group - ΔCt control group, where ΔCt = Ct target gene - Ct reference gene. Ct represents the cycle threshold, or the number of amplification cycles needed for the real-time fluorescence intensity of the reaction to reach a predetermined threshold during the logarithmic phase of amplification. The experiment was repeated thrice. Primer designs are provided in Table S1.

### Western blot

Cellular total proteins were extracted using RIPA lysis buffer (P0013B, Beyotime, Shanghai), and the subsequent supernatant was harvested. The BCA kit (P0028, Beyotime, Shanghai) was employed to quantify the total protein concentration of each sample. Following protein denaturation, samples were stored at -80 °C for subsequent use. Depending on the size of the target protein bands, 8-12% SDS gels were prepared, and equal amounts of protein samples were loaded into each lane for electrophoretic separation. The proteins were then transferred onto a PVDF membrane (1,620,177, BIO-RAD, USA) and blocked with 5% BSA at room temperature for 1 h. The primary antibodies, rabbit anti-CYP24A1 (PA5-21704, 1:1000, Thermo Fisher), TFPI2 (ab186747, 1:1000, abcam, UK), and GADPH (ab181602, 1:10000, abcam, UK) were applied and incubated overnight at 4 °C. Following three washes with 1×TBST for 5 min each at room temperature, HRP-labeled goat anti-rabbit IgG (ab6721, 1:5000, Abcam, UK) was added as a secondary antibody and incubated for 1 h at room temperature. After an additional three washes with 1×TBST for 5 min each, the membrane was immersed in ECL reagent (1,705,062, Bio-Rad, USA) for 1 min at room temperature. Excess liquid was removed, and the membrane was enveloped with plastic wrap, followed by band exposure imaging on the Image Quant LAS 4000 C gel imager (GE, USA). GAPDH served as the internal reference for cellular total protein and cytoplasmic protein. The relative protein expression level was quantified by the ratio of the grey value of the target band to the reference band. The expression levels of various proteins were evaluated, with each set of experiments being performed in triplicate.

### Immunohistochemistry (IHC)

Breast Cancer (BC) tissues, along with adjacent normal tissues, were fixed using 4% paraformaldehyde, followed by paraffin embedding and serial sectioning to a thickness of 4 μm. Subsequently, the sections were baked at 60 °C for 20 min and transitioned through two xylene baths for 15 min each. The tissues were rehydrated using two 5-minute anhydrous alcohol soaks, followed by immersion in 95%, 90%, 80%, and 70% alcohol solutions, each for 10 min. To block endogenous peroxidase, each section was immersed in 3% H2O2 for 10 min at room temperature. Citrate buffer was added, and sections were microwaved for 3 min for antigen retrieval, followed by a 10-minute room temperature pause. After washing thrice with PBS, the sections were blocked using a normal goat serum solution (Shanghai Bioengineering Co., Ltd., China) for 20 min at room temperature. Primary antibodies, rabbit anti-CYP24A1 (PA5-21704, 1:100, Thermo Fisher) and TFPI2 (ab186747, 1:200, abcam, UK), were applied and sections were incubated at 4 °C overnight. After three washes with PBS, sections were incubated with goat anti-rabbit IgG secondary antibody (ab6721, 1:5000, Abcam, UK) for 30 min. SABC (Vector, USA) was added, and the sections were incubated at 37 °C for 30 min. Subsequent to the addition of a DAB coloring kit (P0203, Beyotime Biotechnology, Shanghai), and a 6-minute coloration period, sections were stained with hematoxylin for 30 s, and gently washed with a slow stream of water. The sections were dehydrated through a sequence of 70%, 80%, 90%, 95% ethanol, and anhydrous ethanol each for 2 min and then cleared in xylene for two 5-minute periods. After sealing with neutral resin, sections were visualized under an upright microscope (BX63, Olympus, Japan). The experiment was replicated three times. Positive staining was determined by the presence of brown or yellow in the cytoplasm, observed and counted across five representative high-power fields.

### CCK-8 assay

The cell viability after lentiviral treatment was assessed using a CCK-8 assay kit (K1018, Apexbio, USA). 1 × 10^4^ cells per well were seeded in a 96-well plate (100 µL/well). Subsequently, 10 µL of CCK-8 solution was added at each time point (0 h, 24 h, 48 h, and 72 h), and incubation was performed at 37 °C for 2 h. The absorbance of each well at a wavelength of 480 nm was measured using a microplate reader (Bio-Rad, Hercules, CA, USA). Each experiment was repeated three times.

### Flow cytometry

Cell apoptosis was detected using Annexin V-FITC/PI double staining. Cells were collected 48 h post-transfection, and the cell concentration was adjusted to 1 × 10^6^ cells/mL, followed by fixing with pre-cooled 70% ethanol solution at 4 °C overnight. After being washed twice with PBS, 100 µL of the cell suspension (containing no fewer than 10^6^ cells/mL) was taken and, after two further washes with PBS and centrifugation, cells were resuspended in 200 µL of binding buffer. 10 µL of Annexin V-FITC and 5 µL of PI were gently mixed in, and the mixture was left to react in the dark at room temperature for 15 min. After adding 300 µL of binding buffer, cell apoptosis was assessed using a flow cytometer (Attune NxT, Thermo Fisher, USA) with an excitation wavelength of 488 nm.

### Transwell experiment

In vitro cell migration and invasion assays were conducted using 8 μm-pore sizes Transwell chambers from Corning Incorporated (USA) within a 24-well plate format. Within these 8 μm-pore polycarbonate membrane Transwell chambers, the lower chamber was pre-loaded with 600 mL of DMEM medium containing 20% FBS and allowed to equilibrate at 37 °C for 1 h. MCF-7 cells, post 48-hour transfection, were resuspended in FBS-free DMEM medium and seeded in the upper chamber at a density of 1 × 10^6/mL, followed by incubation at 37 °C, 5% CO2 for 24 h. Subsequently, the Transwell chambers were washed twice with PBS, each time for 5 min, then fixed with 4% paraformaldehyde for 20 min, followed by three washes with PBS, each for 5 min. Afterward, cells were stained with 0.1% crystal violet for 10 min and washed three times with PBS, each time for 5 min. Surface cells were removed using a cotton swab, and the cells that had migrated through the Transwell chambers were observed under an inverted fluorescence microscope (Nikon TE2000, Japan), photographed in 5 random fields of view, and counted. The average cell number that migrated through the Transwell chambers was recorded for each group. Each experiment was repeated three times.

### Nude mice tumor formation and metastasis experiment

Six-week-old BALB/c nude mice (Chinese Academy of Medical Sciences & Peking Union Medical College, Beijing, China) were caged and housed in an SPF-grade animal laboratory with a humidity of 60 − 65% and a temperature of 22–25 °C. They were provided with free access to food and water under a 12-hour light and dark cycle and allowed to adaptively feed for one week before the commencement of the experiment. The experimental procedures and animal usage plan have been approved by the Animal Ethics Committee.

A number of 2 × 10^5^ MCF-7 cells/0.2 mL (including oe-NC, oe-CYP24A1, and oe-TFPI2 variations) were respectively injected subcutaneously into the left axilla of the nude mice, and prior to inoculation, the cells were premixed with Matrigel gel (Solebao YZ-356234-5 ml) to prepare the respective cell suspensions. The nude mice were randomly divided into 3 groups (6 per group): (1) oe-NC group: injected with HCC70 cells transfected with oe-NC; (2) oe-CYP24A1 group: injected with MCF-7 cells transfected with oe-CYP24A1; (3) oe-TFPI2 group: injected with MCF-7 cells transfected with oe-TFPI2. Tumor sizes were measured from day 5 post-injection, with measurements taken every 5 days to record tumor growth, and after 30 days, tumors from each group of nude mice were harvested for subsequent experiments.

For the metastasis nude mouse xenograft model, the mice were randomly assigned to 3 groups, each consisting of 6 animals. Six weeks after being raised, a breast cancer lung metastasis model was established by injecting cells (2 × 10^5^ cells/0.2 mL) into the nude mice via tail vein injection. Upon completion of the breeding period, the mice were dissected via the cervical spine, and lung tissue was collected for H&E staining to assess lung metastasis.

### H&E staining

Lung tissue samples from each group were first washed with saline, then fixed in 4% paraformaldehyde for 30–50 min, followed by processes of washing, dehydration, clarification, wax immersion, embedding, and sectioning. The tissue sections were flattened and adhered to glass slides, then dried in a 45 °C incubator, followed by deparaffinization and washing for 5 min with progressively diluting alcohol concentrations and distilled water. Hematoxylin staining was performed for 5 min, after which the sections were rinsed under tap water for 3 s, followed by differentiation in 1% hydrochloric alcohol for 3 s and eosin staining for approximately 3 min. Finally, the sections underwent dehydration, clarification, and cover-slipping processes. Tissue sections were observed under a microscope. A total of 18 nude mouse samples were used in this experiment.

### Statistical analysis

Statistical data analysis in this study was conducted using SPSS 21.0 statistical software from IBM. Quantitative data were presented as mean ± standard deviation. Initially, data from cancer tissues and adjacent non-cancerous tissues were tested for normality and homogeneity of variance; if they met the requirements of normal distribution and homogeneity of variance, paired t-tests were used for comparison; unpaired t-tests were used for comparison between the other two groups; one-way analysis of variance (ANOVA) was used for comparison among multiple groups. For comparisons of data at different time points, cell activity was analyzed using two-way ANOVA, while tumor data were analyzed using variance analysis for repeated measures data. A P-value of less than 0.05 was considered to indicate a statistically significant difference.

## Results

### Single-cell RNA-seq analysis revealed the presence of a large number of highly variable genes in BC tissues

**Fig. 1:**
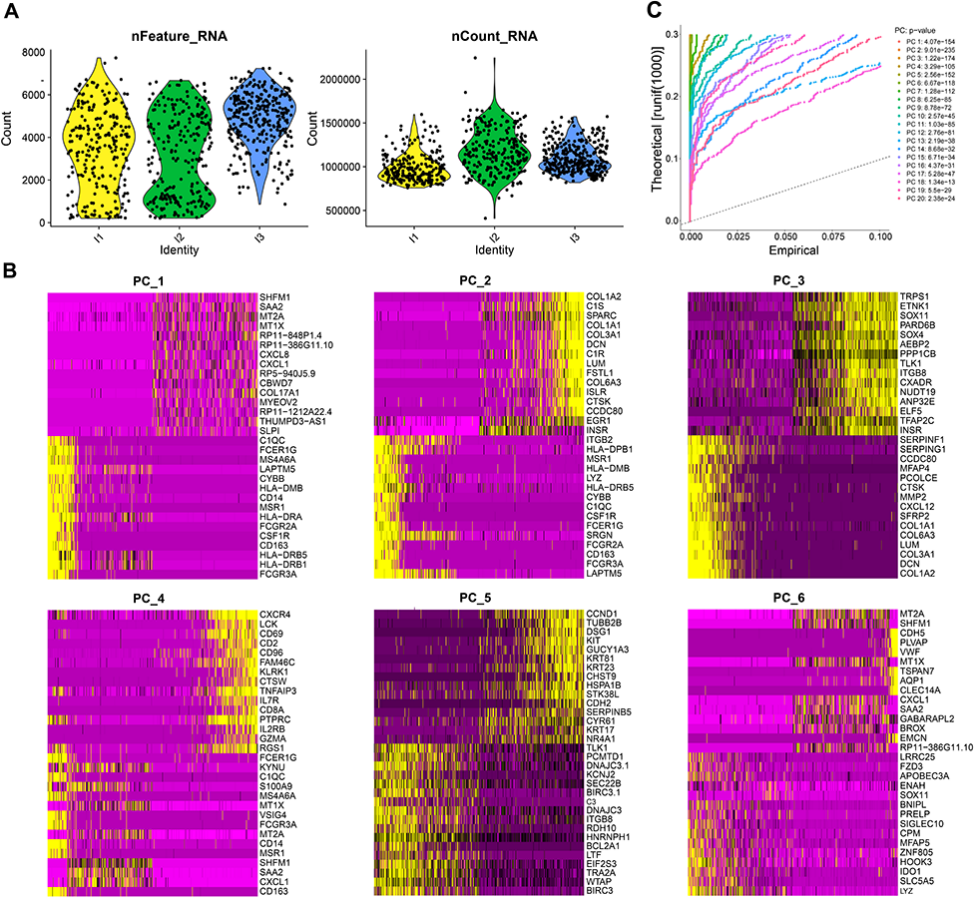
Differential expression genes in BC tissue samples analyzed by single-cell RNA-seq. *Note*: (**A**) Quality control of 1534 cells from BC tissue samples; (**B**) Expression heat figure of the constituent genes in the first 6 principal components before PCA analysis; (**C**) JackStraw plot, where different colored lines represent different principal components, and the annotation in the upper right corner represents the p-value of the principal components

We downloaded BC single-cell RNA-seq data (GSE118389) from the Gene Expression Omnibus (GEO) database. The chip data samples of GSE118389 are derived from tumor tissues of 6 BC patients. The sequencing libraries were constructed using the optimized Smart-seq2 method and sequenced on Illumina NextSeq 500. Here, we have downloaded the raw single-cell expression matrices of 1534 cells provided by the GEO database. The Seurat package in R software performed quality control on the single-cell sequencing data, filtering out low-quality cells (Fig. 1A). By analyzing the filtered cells, we select the top 2000 genes with high variability in expression for subsequent analysis.

When exploring the primary sources of heterogeneity in the dataset, we use the function DimHeatmap and try to determine which principal components (PCs) to select for subsequent analysis. Both cells and genes are ranked based on their PCA scores, and here we show the top 6 principal components (Fig. 1B). We have used the JackStraw program to conduct heuristic resampling tests. More specifically, it involves randomly permuting a portion of the data (default is 1%), re-running PCA to construct “null distribution” feature scores, and repeating this process. We consider the PCs with low p-values as “important” PCs and use the JackStrawPlot function to visualize the first 20 PCs, comparing the positional differences between the p-value distribution and the mean distribution of each PC. Generally speaking, the p-value of a “significant” PC is small, i.e., above the dashed line but below the solid line (Fig. 1C). We can see that the top 20 PCs we selected are all above the dashed line, indicating that they are all essential principal components and can fully reflect the information contained in the previous 2000 highly variable genes. We can select these 20 PCs based on the results above for further analysis.

### tSNE clustering analysis and cell annotation of BC organization’s single-cell RNA-seq data

**Fig. 2:**
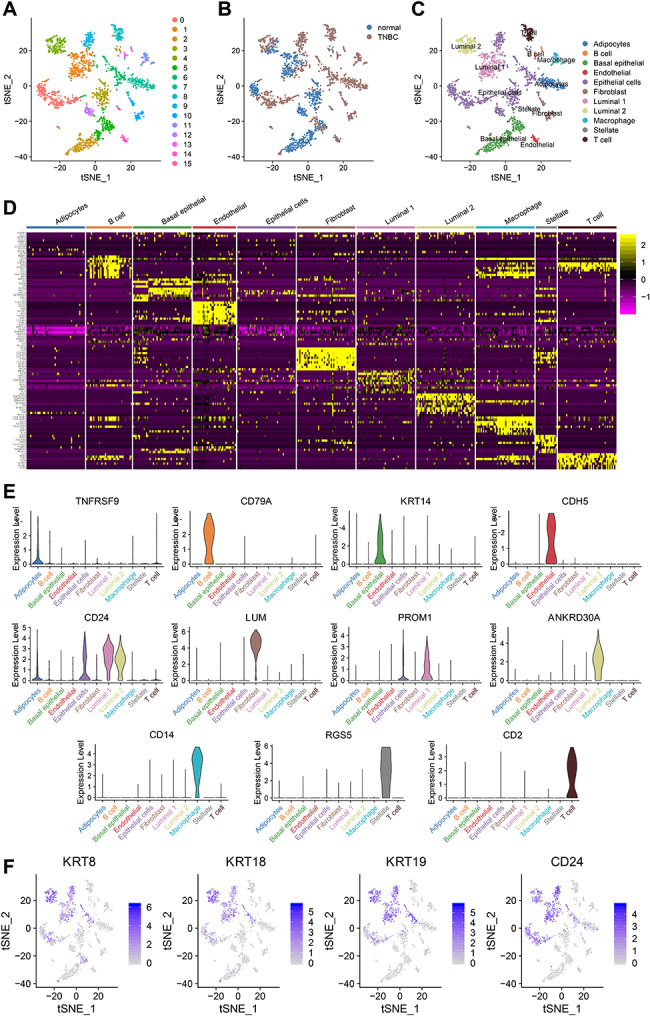
t-SNE clustering analysis and cell annotation of single-cell RNA-seq data from BC tissue samples. *Note*: (**A**) tSNE clustering analysis clusters cells into 16 cell clusters; (**B**) The sample sources of tSNE clustered cell clusters; (**C**) 24 cell clusters annotated as 11 cell types; (**D**) Heatmap of marker gene expression in cell clusters; (**E**) Violin plots of marker gene expression in cell clusters; (**F**) Scatter plot of expression of luminal epithelial cells in cell clusters

t-SNE (t-Distributed Stochastic Neighbor Embedding) combines dimensionality reduction (e.g., PCA) with random walks on the nearest neighbor graph to map high-dimensional data into a two-dimensional space while preserving local distances between points. Compared to PCA, t-SNE is a stochastic algorithm, which means that running the method multiple times on the same dataset will result in different figures. Therefore, we use the tSNE method to reduce further and visualize the data’s dimensionality. Using cluster analysis, we categorized all cells into 16 clusters (Fig. 2A). After adjusting for batch effects, there were no significant differences between cells from different sample sources within the cell clusters, only differences in the proportions of cell clusters (Fig. 2B).

Known cluster marker genes include SLPI, PROM1, KRT19 (Luminal 1 type; [15]; ANKRD30A, SYTL2 (Luminal 2 type; [15]; KRT14, KRT5, ACTA2, MYLK, TP63, ITGA6, KRT17, MME (basal epithelial cells; [15, 16]; RGS5, ACTA2, PDGFRB, ADIRF (stellate cells; [17]; LUM, DCN, COL1A1 (fibroblasts; [17]; PECAM1, VWF, CDH5, SELE, PLVAP, CLDN5 (endothelial cells; [16, 17]; CD2, CD3D, CD3E, CD3G, CD8A, CD8B, CD4 (T cells; [16, 17]; MS4A1, CD79A, CD79B, BLNK, CD52 (B cells; [16, 17]; CD14, CD68, CD163, CSF1R, AIF1 (macrophages; [17]. We used the “SingleR” package from Bioconductor/R software to annotate these 16 cell clusters, resulting in annotation into 11 cell types (Fig. 2C).

Furthermore, we generated an expression profile of BC cell-specific marker genes (Fig. 2D) and identified clustered cell populations based on the known marker genes of each cell type (Fig. 2E). We use known cell type marker genes to identify cell clusters: PROM1 is a specific marker gene for L1 cell clusters, ANKRD30A is a specific marker gene for L2 cell clusters, KRT14 is a specific marker gene for basal epithelial cells, CD14 is a specific marker gene for macrophage clusters, CDH5 is a specific marker gene for endothelial cells, LUM is a specific marker gene for fibroblasts, RGS5 is a specific marker gene for stellate cells, CD79A is a specific marker gene for B cells, CD2 is a specific marker gene for T cells, and TNFRSF9 is a specific marker gene for adipocytes. Next, we examined the expression of specific marker genes (KRT8, KRT18, KRT19, and CD24) in cell clusters of BC tissue samples to confirm the suitability of our cell clustering based on known cell marker genes (Fig. 2F).

### There are differences in the number of differentially expressed genes and biological functions in the luminal epithelial cells of 1/2-type luminal cells in BC tissue samples

**Fig. 3:**
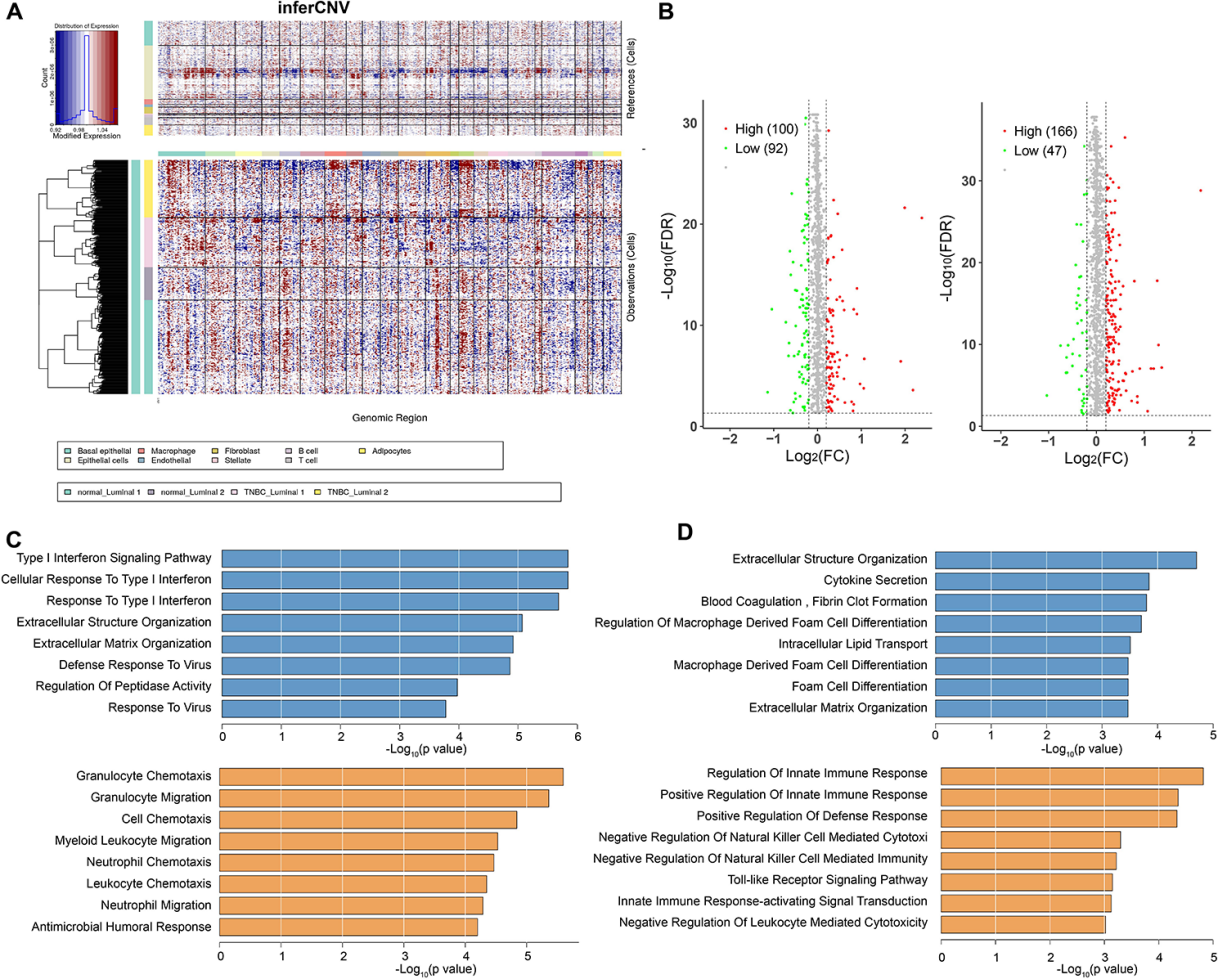
The CNV analysis and GO enrichment analysis of single-cell RNA-seq sequencing data from BC tissues. *Note*: (**A**) Inference of copy number variations in single-cell RNA-seq sequencing data of BC tissue. (**B**) Scatterplot figure showing differentially expressed genes in BC tissue compared to normal breast tissue in luminal epithelial cells. (**C**) GO enrichment analysis of differentially expressed genes in type 1 luminal epithelial cells. (**D**) GO enrichment analysis of differentially expressed genes in type 2 luminal epithelial cells

It is worth noting that although we have identified two types of luminal epithelial cells in normal breast and BC tissue, they are not clustered in the Figure. To define malignant cells in luminal epithelial cells, we performed copy number analysis using the “inferCNV” package in R software and clustered copy number variations using unsupervised clustering algorithms to identify copy number variation patterns in tumor cells derived from different breast cancer samples. As shown in (Fig. 3A), we can see that tumor cells from the same BC tissue sample are dispersed into multiple distinct clusters, indicating the presence of differences in malignancy among tumor cells originating from the same BC sample.

Next, we analyzed the differential gene expression of type 1 luminal epithelial cells between BC tissue and normal breast tissue and identified 166 upregulated genes and 47 downregulated genes (Fig. 3B); for type 2 luminal epithelial cells, we found 100 upregulated genes and 92 down-regulated genes in BC tissue compared to normal breast tissue (Fig. 3B). In further exploring the functional analysis of type 1 and type 2 luminal epithelial cells, it was observed that the upregulated genes in BC tissue primarily involve the Type I interferon signaling pathway, cellular response to Type I interferon, response to Type I interferon, and regulation of peptidase activity. On the other hand, the downregulated genes are mainly associated with granulocyte chemotaxis, granulocyte migration, cell chemotaxis, bone marrow leukocyte migration, neutrophil chemotaxis, and neutrophil migration (Fig. 3C). However, upregulated genes in type 2 tubular epithelial cells mainly involve extracellular structural organization, cytokine secretion, macrophage-derived foam cell differentiation regulation, intracellular lipid transport, foam cell differentiation, and extracellular matrix organization. Downregulated genes mainly regulate innate immune response, negative regulation of natural killer cell-mediated cytotoxicity, immune suppression mediated by natural killer cells, Toll-like receptor signaling pathway, and leukocyte-mediated cytotoxicity (Fig. 3D).

The above results indicate that there are differences despite tumor cells from the same BC sample source, and there are also differences in the expression levels of differential genes and biological functions between type 1 and type 2 luminal epithelial cells.

### The risk assessment model based on the CYP24A1 and TFPI2 genes for predicting the prognosis of BC patients has good accuracy

**Fig. 4:**
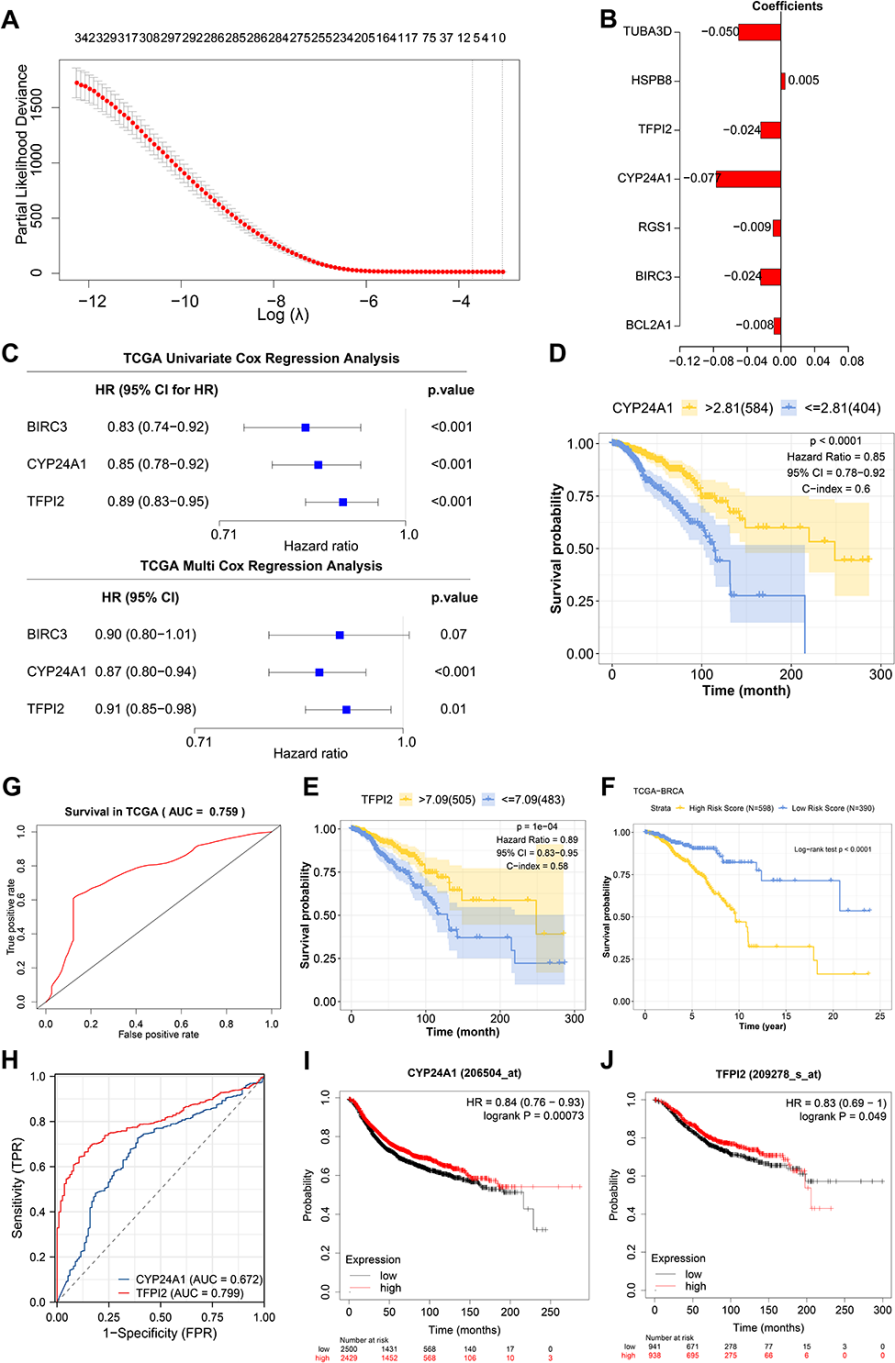
Univariate and multivariate Cox regression analysis for screening prognostic molecular markers and constructing a risk assessment model in breast cancer (BC). *Note*: (**A**) Lasso regression coefficient distribution; (**B**) Regression equation coefficients of 7 prognostic-related factors selected by Lasso regression; (**C**) Forest Figure visualizing univariate Cox analysis and multivariate Cox analysis of TCGA-BRCA cohort data; (**D**–**E**) Kaplan-Meier survival curves of CYP24A1 and TFPI2 genes; (**F**) Overall survival curve of TCGA-BRCA cohort patients grouped by Risk Score using Kaplan-Meier; (**G**) ROC curve depicting the predictive performance of the risk assessment model on TCGA-BRCA cohort; (**H**) ROC curve demonstrating the accuracy of the prognostic model constructed by gene expression of CYP24A1 and TFPI2 in predicting BC patients’ prognosis; (**I**) Kaplan-Meier survival curve of CYP24A1 gene; (**J**) Kaplan-Meier survival curve of TFPI2 gene

First, we used the TCGA-BC dataset to select BC patient samples, filtering out samples without survival data, and performed Lasso regression. Seven genes related to the prognosis of BC patients were screened out from 405 differentially expressed genes (Fig. 4A-B). Next, we conducted a univariate Cox regression analysis. To reveal the differentially expressed genes (DEGs) and clinical features (Fig. 4C) significantly associated with the prognosis of BC patients. For genes and clinical features with a log-rank P-value < 0.05, we further conducted multivariable Cox regression analysis to identify genes (CYP24A1 and TFPI2) that were associated with prognosis (Fig. 4C). Ultimately, we obtained two genes (CYP24A1 and TFPI2) associated with prognosis. Survival analysis was performed using Kaplan-Meier curves, and the results revealed a significant correlation between CYP24A1 and TFPI2 with the prognosis survival time of breast cancer patients (Fig. 4D-E). Based on the results of above analysis, we have established a risk assessment model to predict the prognosis of BC patients: Risk Score = (-0.156 * CYP24A1) + (-0.115 * TFPI2). We used the model to calculate the risk score, survival analysis, and ROC curve analysis results from the TCGA-BRCA sample cohort. The results showed that patients with a low-risk score had significantly longer overall survival than those with a high-risk score.

Additionally, the AUC of the risk assessment model for predicting 10-year overall survival in BC patients was 0.759 (Fig. 4F-G). We observed a significant difference in overall survival between the two groups by performing survival analysis using the KMplot website. Validation of the GEO dataset revealed a significant correlation between the expression of CYP24A1 and TFPI2 genes and the survival status of breast cancer patients (Fig. 4I-J). The risk assessment model based on CYP24A1 and TFPI2 genes demonstrated good accuracy in predicting the prognosis of BC patients (Fig. 4H).

### Overexpression of CYP24A1 or overexpression of TFPI2 can significantly inhibit the malignant biological behavior of BC cells

**Fig. 5:**
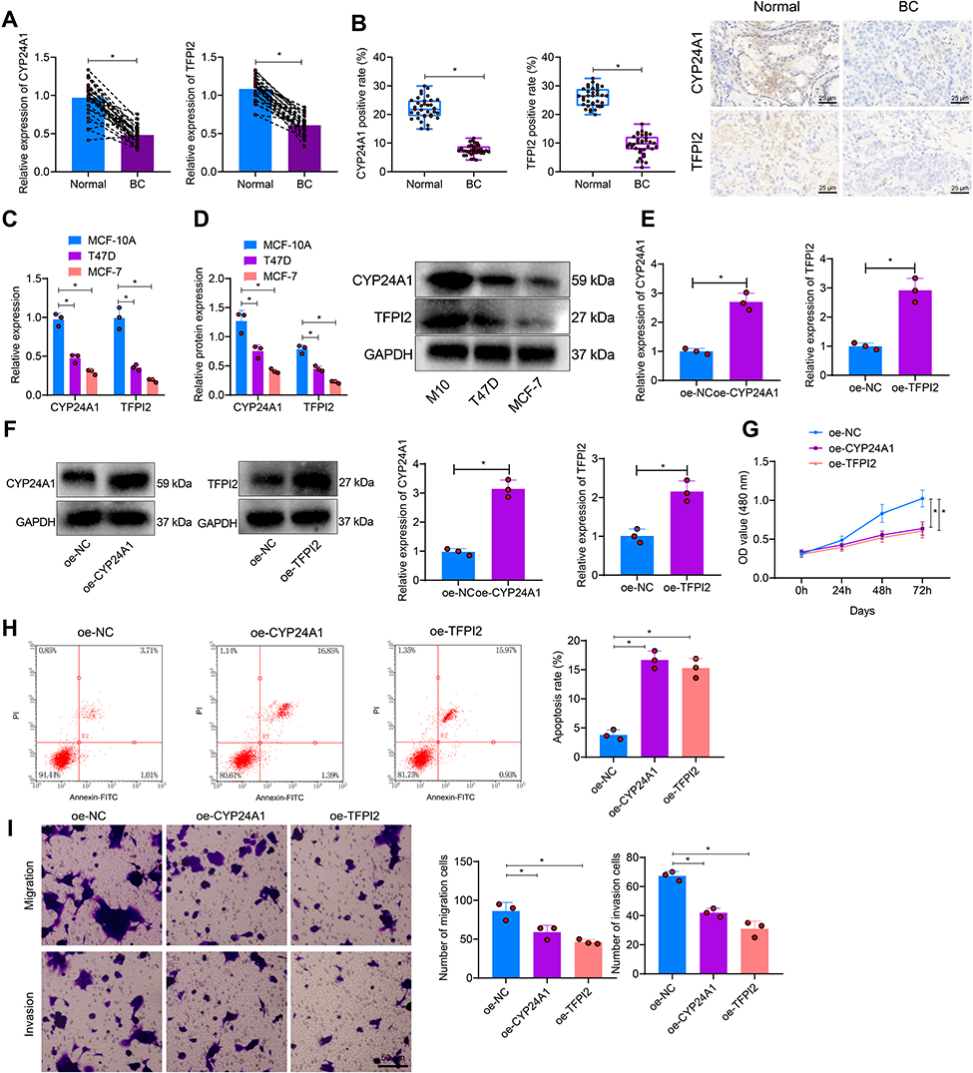
The effects of overexpression of CYP24A1 or TFPI2 on the proliferation, migration, invasion, and apoptosis of BC cells. *Note*: (**A**) Expression of CYP24A1 and TFPI2 in BC tumor tissue and adjacent normal tissue was detected by RT-qPCR (*N* = 35). (**B**) Expression of CYP24A1 and TFPI2 in BC tumor tissue and adjacent normal tissue was detected by IHC (400×, scale bar = 25 μm, *N* = 35). (**C**) mRNA expression levels of CYP24A1 and TFPI2 in regular human breast epithelial cell lines (MCF-10 A) and luminal BC cell lines (MCF-7 and T47D) were detected by RT-qPCR. (**D**) Protein expression of CYP24A1 and TFPI2 in regular human breast epithelial cell lines (MCF-10 A) and luminal BC cell lines (MCF-7 and T47D) was detected by Western blot. (**E**–**F**) RT-qPCR and Western blot detected the infection efficiency of overexpressed CYP24A1 and TFPI2 lentivirus in MCF-7 cells. (**G**) Cell proliferation of MCF-7 cells was assessed by CCK8 assay. (**H**) Cell apoptosis of MCF-7 cells was detected by flow cytometry. (**I**) Cell migration and invasion of MCF-7 cells were examined by Transwell assay. The data in Figure are all measurement data, represented by sample mean ± standard deviation; paired t-test is used for Comparison between cancer tissue and adjacent normal tissue, independent sample t-test is used for Comparison between two groups, and one-way analysis of variance is used for Comparison between multiple groups Comparison of inter-group data at different time points, cell viability was analyzed using a two-way analysis of variance. * represents the Comparison between two groups, with *P* < 0.05

To validate the effects of two prognostic-related genes, CYP24A1 and TFPI2, obtained through differential gene expression analysis in BC luminal epithelial cells and TCGA database analysis on luminal breast cancer, we first detected the expression of CYP24A1 and TFPI2 in BC tumor tissues and adjacent normal tissues using RT-qPCR and immunohistochemistry (IHC). The results showed that compared to the normal tissue surrounding cancer, CYP24A1 and TFPI2 were significantly decreased in BC cancer tissue (Fig. 5A-B). In addition, we further utilized RT-qPCR and Western blot to detect the expression of CYP24A1 and TFPI2 in normal human breast epithelial cells (MCF-10 A) and luminal-type BC cell lines (MCF-7 and T47D). The results showed that compared to the MCF-10 A cell line, the expression of CYP24A1 and TFPI2 in the two BC cell lines was significantly decreased, and in the MCF-7 cell line, the expression was lower. Therefore, we chose the MCF-7 cell line for further experiments (Fig. 5C-D).

We overexpressed CYP24A1 and TFPI2 in the MCF-7 cell line and assessed the overexpression efficiency by RT-qPCR and Western blot analysis of lentivirus-infected cells. The results showed that after overexpression of CYP24A1, the mRNA and protein expression of CYP24A1 in the cells significantly increased (Fig. 5E-F). Similarly, after overexpression of TFPI2, the mRNA and protein expression of TFPI2 in the cells also significantly increased (Fig. 5E-F).

Next, we used CCK-8 to detect cell proliferation, flow cytometry to detect cell apoptosis, and the Transwell assay to evaluate cell migration and invasion ability. Compared to the oe-NC group, BC cells’ proliferation, migration, and invasion abilities were significantly reduced in the oe-CYP24A1 and oe-TFPI2 groups. Additionally, cell apoptosis was significantly increased (Fig. 5G-I).

### Overexpression of CYP24A1 or TFPI2 can inhibit BC cells’ tumorigenic and metastatic abilities

**Fig. 6:**
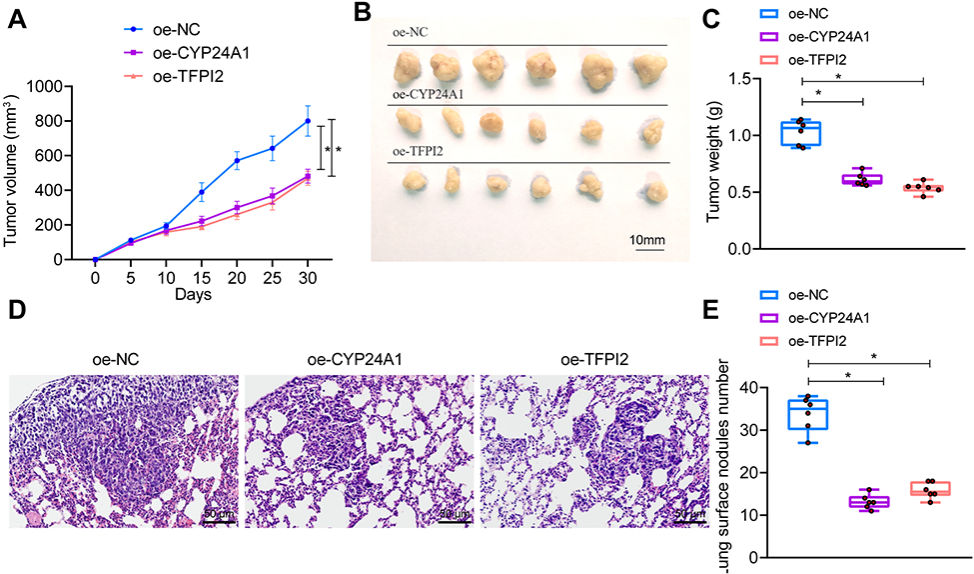
The effect of CYP24A1 or TFPI2 overexpression on tumorigenic and metastatic abilities in BC cells. *Note*: (**A**) Growth curves of tumor masses in nude mice in each group; (**B**) Representative Figures of tumor masses in nude mice in each group; (**C**) Comparison of tumor mass weights in nude mice in each group; (**D**) Observation of lung metastasis of tumor masses in nude mice in each group through H&E staining (10×, scale bar = 1 cm.); (**E**) Counting of lung metastatic nodules in nude mice in each group. The data in Figure are all metric data, represented by sample mean ± standard deviation. Single-factor analysis of variance is used for Comparison among multiple groups. Comparison of data between different groups at different time points is conducted using repeated measures analysis of variance. * represents the Comparison between two groups, with *P* < 0.05. *N* = 6

To further investigate the effects of CYP24A1 and TFPI2 on the tumorigenic capacity of breast cancer cells in vivo, we established a nude mouse xenograft animal model for breast cancer transplantation. After 30 days, we measured the volume of the tumor tissue and removed the tumor tissue for weighing. The results showed that compared with the oe-NC group, the tumor volume and weight in the oe-CYP24A1 group and the oe-TFPI2 group were significantly reduced (Fig. 6A-C).

To investigate the effects of CYP24A1 and TFPI2 on tumor metastasis, we stably transfected BC cells and injected them into nude mice via the tail vein. The metastasis of lung tumors in each group of nude mice was observed through H&E staining. The results showed that compared to the oe-NC group, the tumor metastatic nodules in the lungs of the oe-CYP24A1 and oe-TFPI2 groups were significantly reduced (Fig. 6D-E).

These results indicate that overexpression of CYP24A1 or TFPI2 can suppress BC cells’ tumorigenic and metastatic capabilities in vivo.

## Discussion

In light of burgeoning developments in bioinformatics and molecular biology, models assessing risk based on gene expression have accrued mounting clinical pertinence, especially in the sphere of breast cancer, which stands as the most predominant malignancy impacting women globally [18]. This research aimed to elucidate the roles of CYP24A1 and TFPI2 genes in breast cancer and to formulate a predictive model for risk assessment with robust characteristics.

Preliminary research into CYP24A1 has subtly indicated its linkage with several tumor types, though its precise role and implications in breast cancer have lingered in a somewhat nebulous state [19]. Our work seeks to underscore the crucial involvement of CYP24A1 in breast cancer, providing novel insights into its probable therapeutic ramifications. Concurrently, while TFPI2 has previously been associated with tumor genesis and prognosis in various cancers, its exact role and prognostic value in breast cancer have largely remained underexplored [20, 21]. This study endeavors to shed light on this association, establishing a foundational platform for subsequent studies concerning TFPI2 and breast cancer.

The predictive model introduced in our study presents several commendable merits in accuracy and applicability compared to extant prognostic models for breast cancer. The superior resolution and enhanced sensitivity afforded by single-cell RNA-seq technology substantively contribute to these merits [22–24]. Nonetheless, it is paramount to acknowledge the intrinsic limitations of every model, as underscored by the fact that our model primarily hinges on samples from Chinese breast cancer patients, potentially limiting its generalizability across varied populations and ethnic groups [24]. Although our study utilized the scRNA-seq data from the GSE118389 dataset of ductal breast cancer (BC) samples to investigate cellular heterogeneity, we acknowledge the existence of multiple scRNA-seq datasets in the field of breast cancer research. We chose GSE118389 due to its direct relevance to ductal breast cancer and its importance in terms of cellular diversity and high-quality sequencing data, which are crucial for analyzing cellular heterogeneity, gene expression, and biological functions of breast cancer. However, this choice also has limitations. Specifically, regarding the prognostic markers CYP24A1 and TFPI2, our focus was solely on GSE118389, which raises concerns about the generalizability of our findings. Although these markers have shown promise in analysis and experimental validation, their applicability in a broader population and different breast cancer backgrounds needs further verification. To address this limitation, we plan to expand our analysis to include multiple scRNA-seq datasets encompassing different subtypes of breast cancer. This will help validate the prognostic relevance of CYP24A1 and TFPI2 in a more diverse range of breast cancer cases, thus enhancing the generalizability and clinical utility of our findings. In conclusion, while our study provides valuable insights into cellular heterogeneity and potential therapeutic targets for ductal breast cancer, the reliance on a single dataset underscores the need for further research using more diverse datasets to ensure broader applicability and validation of the prognostic markers we have identified.

Navigating through the complexity of breast cancer prognosis presents a formidable challenge, illustrating the difficulty in forging a singular model that accommodates the variegated needs of all patient demographics [25]. Prospectively, integrating several models or biomarkers, which might encompass proteins and metabolites, could potentially construct a more exhaustive and clinically applicable prognostic framework [26]. This advancement will likely demand an intensification of interdisciplinary collaboration and an augmentation of validation research initiatives [27].

Further scientific exploration into the biological mechanics regulated by CYP24A1 and TFPI2 is imperative, focusing particularly on their impact on breast cancer cell proliferation, metastasis, and therapeutic responsiveness [19, 28]. Furthermore, probing into the potential for deploying these two genes as innovative therapeutic targets, modulating their expression or activity via specific drugs or approaches, beckons significant promise in amplifying the efficacy of breast cancer therapeutic strategies [29–31].

## Conclusion

**Fig. 7:**
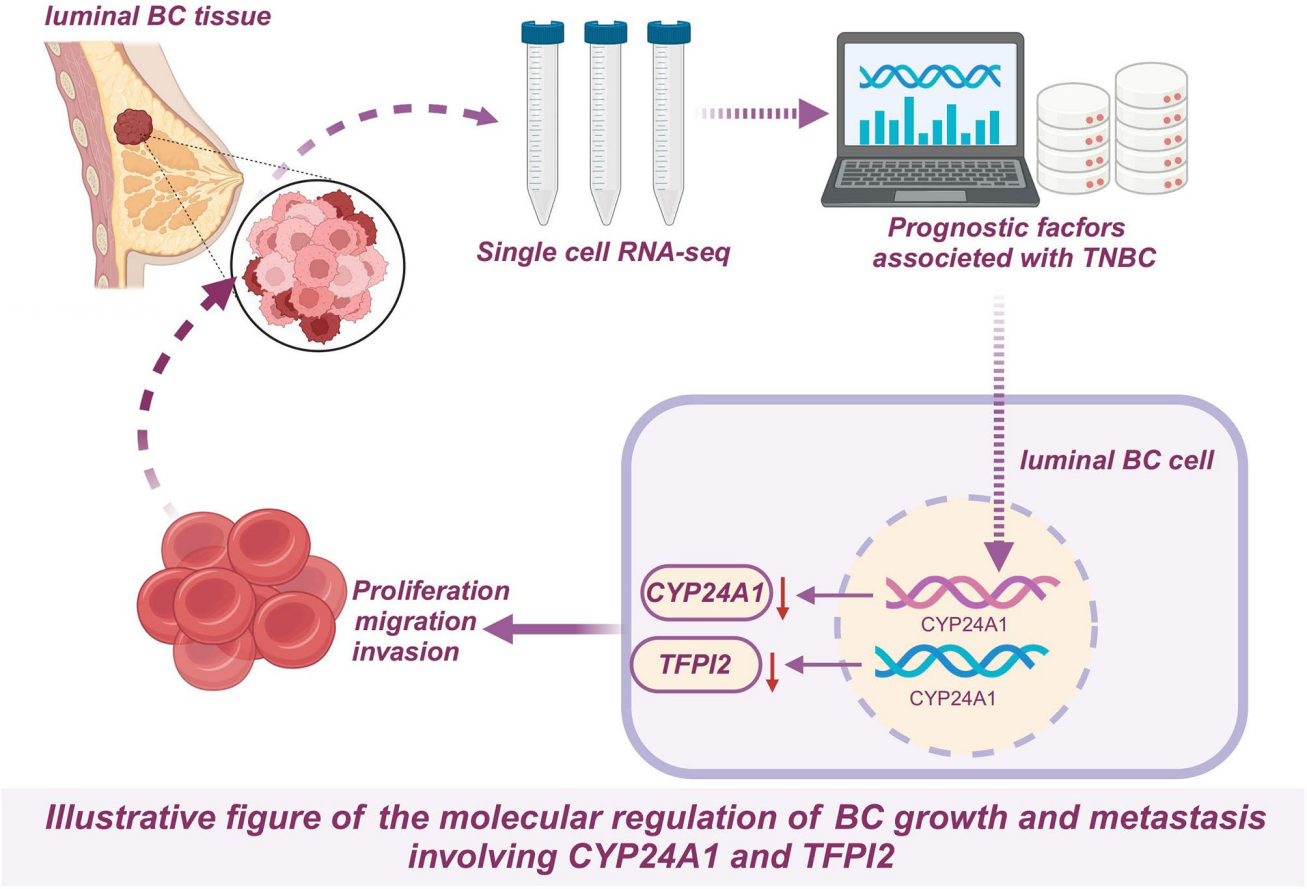
The molecular regulation of CYP24A1 and TFPI2 in the growth and metastasis of breast cancer

In conclusion, this research has adeptly uncovered pivotal roles that CYP24A1 and TFPI2 undertake in breast cancer, and has established a predictively precise risk assessment model. This not only carves out new pathways for subsequent research and treatment in breast cancer but also proffers pragmatic value to clinical applications, thereby enhancing the potential for improved patient outcomes (Fig. 7).

### Electronic supplementary material

Below is the link to the electronic supplementary material.

Supplementary Table 1

Supplementary Figure 1

## Data Availability

The datasets generated and/or analyzed during the current study are available from the corresponding author on reasonable request.

## Abbreviations

BC: Breast cancer
CYP24A1: 25-hydroxyvitamin D-24-hydroxylase
TFPI2: Tissue factor pathway inhibitor 2
ER-negative BC: Estrogen receptor-negative BC
ERK1/2: Extracellular signal-regulated kinase 1/2
GEO: Gene Expression Omnibus
UMI: Unique molecular identifier
CNV: Single-cell copy number variation
HR: Hazard ratio
cDNA: Complementary DNA
RT-qPCR: Reverse transcription-quantitative polymerase chain reaction
GAPDH: Glyceraldehyde-3-phosphate dehydrogenase
BCA: Bicinchoninic acid
ANOV: Analysis of variance
