# Supplementary material for: Unveiling heterogeneity and prognostic markers in ductal breast cancer through single-cell RNA-seq
Source: Cancer Cell Int. 2024 Jul 27;24:266. doi: 10.1186/s12935-024-03325-1 (PMC11282761; doi:10.1186/s12935-024-03325-1)
Supplement: Supplementary file 1 — Supplementary Table 1 [file 12935_2024_3325_MOESM1_ESM.docx]

**Table S1** Primer sequences

| Gene | Sequences (5’-3’) |
| --- | --- |
| CYP24A1 | Forward: AAAGTATCTGCCTCGTGTTGTA |
|  | Reverse: CTTCTCTAACCGGTTGTCGATA |
| TFPI2 | Forward: TACAGTCCAAAAGATGAGGGAC |
|  | Reverse: GAATTTTCCGGATTCTACTGGC |
| GAPDH | Forward: ACAACTTTGGTATCGTGGAAGG |
|  | Reverse: GCCATCACGCCACAGTTTC |
