# Supplementary figures and images for: Unveiling heterogeneity and prognostic markers in ductal breast cancer through single-cell RNA-seq
Source: Cancer Cell Int. 2024 Jul 27;24:266. doi: 10.1186/s12935-024-03325-1 (PMC11282761; doi:10.1186/s12935-024-03325-1)

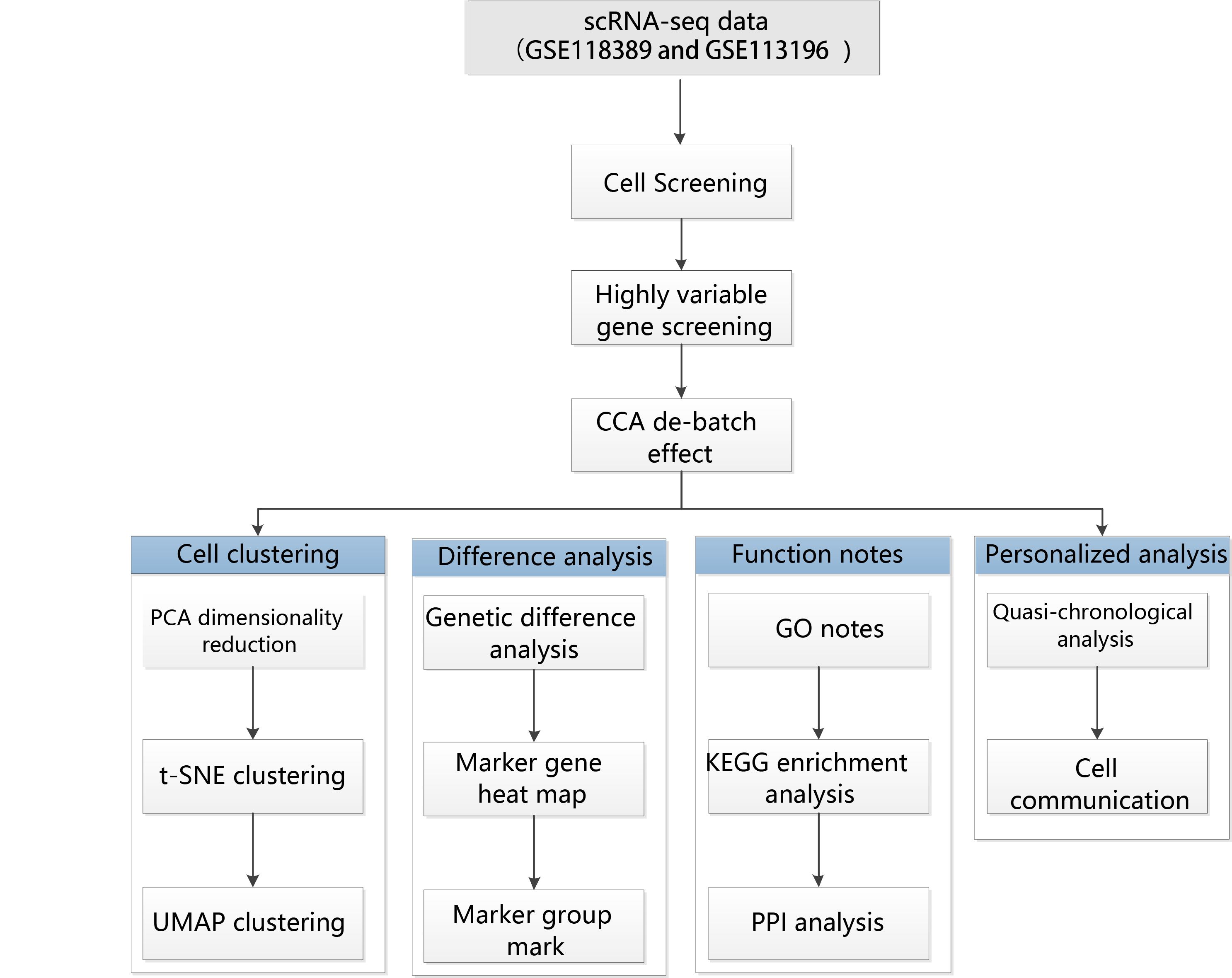

Supplement: Supplementary file 2 — Supplementary Figure 1 [file 12935_2024_3325_MOESM2_ESM.jpg]
